# An introduction to PIWI-interacting RNAs (piRNAs) in the context of metazoan small RNA silencing pathways

**DOI:** 10.1080/15476286.2022.2132359

**Published:** 2022-10-10

**Authors:** Astrid D. Haase

**Affiliations:** National Institutes of Diabetes and Digestive and Kidney Diseases, National Institutes of Health, Bethesda, MD, USA

**Keywords:** piRNA, PIWI, RNAi

## Abstract

PIWI proteins and their associated PIWI-interacting RNAs (piRNAs) constitute a small RNA-based adaptive immune system that restricts the deleterious activity of mobile genetic elements to protect genome integrity. Self/nonself discrimination is at the very core of successful defence and relies on complementary base-pairing in RNA-guided immunity. How the millions of piRNA sequences faithfully discriminate between self and nonself and how they adapt to novel genomic invaders remain key outstanding questions in genome biology. This review aims to introduce principles of piRNA silencing in the context of metazoan small RNA pathways. A distinct feature of piRNAs is their origin from single-stranded instead of double-stranded RNA precursors, and piRNAs require a unique set of processing factors. Novel nucleases, helicases and RNA binding proteins have been identified in piRNA biology, and while we are starting to understand some mechanisms of piRNA biogenesis and function, this diverse and prolific class of small RNAs remains full of surprises.

## Introduction

Retroviruses and transposons pose a threat to genome stability [1]. In the ongoing arms race with these mobile genetic elements, host genomes suffered insults, accumulated scars, and in rare instances adopted transposon sequences for their own use [2]. But above all, they established control [3,4]. RNA-guided immunity -CRISPR/Cas and RNA interference pathways- restrict mobile genetic elements to protect genome integrity [5]. Animal germ cells employ a specialized small RNA pathway -PIWI proteins and their PIWI-interacting RNAs (piRNAs)- to ensure genome stability and fertility [4,6]. PIWI-piRNA silencing complexes (piRISC) degrade transposon transcripts in the cytoplasm and establish epigenetic restriction in the nucleus.

PiRNAs are largely confined to germ cells but the initial epigenetic restriction they impose is maintained in adult somatic cells and essential for health. Deterioration of maintenance during ageing and in disease unleashes transposons that trigger toxicity and drive mutagenesis [7,8]. Understanding how transposons are controlled has fundamental implications for reproductive pathologies, age-related diseases, cancer biology and auto-immune disorders, all of which are associated with loss of transposon control [1,9].

Most of our knowledge about piRNA pathways is based on studies in the insect model *Drosophila melanogaster* and valuable complementary ex vivo cultures from fly and silkworm ovaries [10]. Studies in different model organisms and characterization of human piRNAs suggested conserved functions in genome protection and fertility, but also revealed variations in molecular patterns and mechanisms [11–15]. Key features of piRNA pathways allow us to assemble a framework to understand the many different flavours of piRNA biology.

## A short history of RNA interference

RNA silencing pathways have been discovered independently from different angles. In plants, efforts to intensify the colour of petunia flowers using transgene expression observed -against all intentions- a variegating phenotype with heritable loss of pigmentation [16,17]. Similar silencing effects dependent on homologous RNA sequence were observed in fungi and worms [18,19]. Systematic investigations revealed double-stranded RNA as the most efficient trigger to induce homology-dependent gene silencing [20], and Andrew Fire and Craig Mellow received the Nobel prize for what became known as RNA interference (RNAi). MicroRNAs (miRNAs) were identified independently around the same time [21,22]. In C. elegans, the lin-4 gene had long been known as a master regulator of developmental timing, but no protein product could be identified. Lin-4 turned out to produce a short RNA (22 nt) with sequence complementarity to its target lin-14 [23,24]. The emerging realization that small non-coding RNAs silence gene expression revolutionized biomedical research, biotechnology and therapy: miRNAs were uncovered as key regulators of gene expression in development and disease [25], small interfering RNAs (siRNAs) enabled loss-of-function (knock-down) studies in cultured cells [26], and RNA-based therapeutics transformed our thinking about targeted therapies [27].

## The RNA induced silencing complex (RISC)

**Figure 1.**
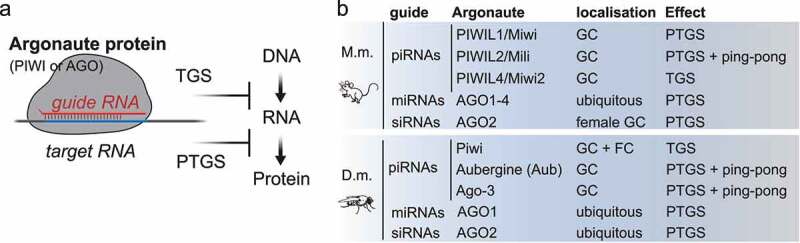
The RNA-induced silencing complex (RISC). (A) Argonaute proteins (AGO and PIWI subfamilies) associate with small RNAs to form RNA-induced silencing complexes (RISC). Within RISC, the sequence of the guide RNA determines target specificity by complementary base-pairing. The associated Argonaute protein, its subcellular localization and co-factors determine effector mechanisms that result in transcriptional (TGS) or post-transcriptional gene silencing (PTGS). (B) *Mouse (M.m.)* piRNAs associate with PIWIL1-4 in germ cells (GC). PIWIL1-2 piRNA complexes localize to the cytoplasm and induce target-RNA degradation. PIWIL4 piRNA complexes establish epigenetic silencing in the nucleus. microRNAs (miRNAs) associate with AGO1-4. SiRNAs associate with AGO2 in female germ cells. *Drosophila melanogaster (D.m.)* piRNAs associate with one of three PIWI proteins. Piwi-piRISC are present in germ cells and in follicle cells (FC) of the ovary and induce transcriptional silencing (TGS). Aubergine (Aub) and Ago-3 are restricted to germ cells, degrade target RNAs, and engage in ping-pong production of secondary piRNAs. MiRNAs associate with AGO1. SiRNAs associate with AGO2.

At the centre of all small RNA silencing pathways resides the RNA induced silencing complex (RISC), which at its core consists of an Argonaute protein and its associated guide RNA (Figure 1) [28]. Within RISC, the sequence of the small RNA determines target-specificity by complementary base-pairing, and its Argonaute protein partner determines effector mechanisms resulting in transcriptional or post-transcriptional silencing [28]. Argonaute proteins are named after the first phenotype described in *Arabidopsis thaliana* that resulted in small squid-like plants, hence ‘argonaute’ [29], and are conserved from bacteria to humans [30]. The protein family is defined by the presence of a PAZ (Piwi-Argonaute-Zwille) and a PIWI domain, which assumes an RNase H fold and confers ‘slicer’ nuclease activity to Argonautes [31]. In animals, different subfamilies can be distinguished. The AGO-subfamily, similar to A. thaliana Ago-1, is ubiquitously expressed and associates with siRNAs and miRNAs. In contrast, the PIWI-clade of Argonaute proteins, named after *Drosophila* piwi (P-element induced wimpy testes), is mostly restricted to germ cells and associates with piRNAs [4,32,33]. A third subfamily was identified in nematodes and termed ‘worm specific AGOs (WAGOs)’ [34]. Animal genomes encode varying numbers of Argonaute proteins. C. elegans encodes 27 family members that can be classified into one PIWI, two AGOs and 24 WAGOs. *Drosophila* contains two AGOs and three PIWIs, and mammals contain four AGOs (AGO 1–4) and four PIWIs (PIWIL1-4). The function of mammalian PIWI proteins and their associated piRNAs is best described in male germ cells, because our most commonly used mammalian model organisms, mice and rats, have lost the requirement for piRNA silencing in the female germline due to an interesting evolutionary variation [35]. This variation emphasizes the importance of additional model organisms for our understanding of mammalian PIWI-piRNA pathways, and germ cell biology [13–15].

## Small RNA biogenesis in flies and mammals

**Figure 2.**
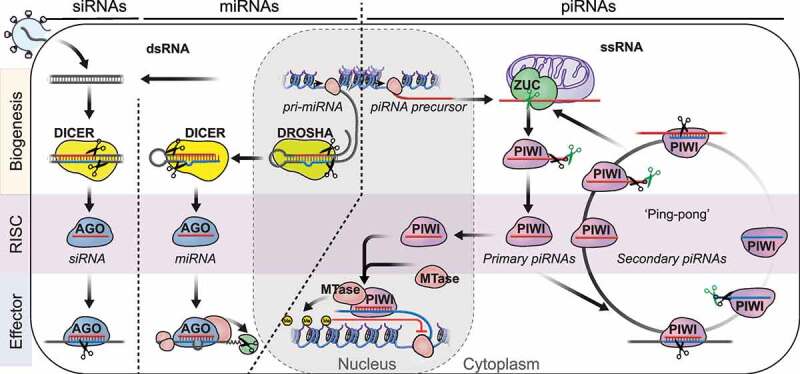
An oversimplified depiction of the three major small RNA pathways in animals. Small interfering RNAs (siRNAs) originate from long double stranded RNA (dsRNA) and are generated by the RNase III enzyme Dicer. SiRNAs associate with AGO-clade Argonaute proteins and degrade target RNA using the slicer activity of Argonaute proteins. MicroRNAs (miRNAs) originate from partly double-stranded RNA hairpins. MiRNA biogenesis proceeds in two steps involving the RNase III enzymes DROSHA and DICER. MiRNAs associate with AGO-clade Argonaute proteins and recruit RNA-degradation machinery to silence their targets post-transcriptionally (PTGS). PIWI-interacting RNAs (piRNAs) originate from long single-stranded precursors. Their biogenesis involves the endonuclease Zucchini/PLD6 (ZUC) (primary piRNAs), or piRNA-guided slicing during ping-pong (secondary piRNAs). Maturation of some piRNAs involves additional 3’ trimming. PIWI-piRNA complexes degrade target-RNA in the cytoplasm or establish lasting epigenetic restriction in the nucleus.

Formation of a functional RNA-induced silencing complex (RISC) complex starts with small RNA biogenesis (Figure 2) [36]. SiRNAs and miRNAs originate from perfect or partly double-stranded RNA substrates and are generated by RNase III enzymes. SiRNAs originate from exogenous (viral) or endogenous double-stranded (ds)RNAs and are generated by the RNase III enzyme DICER [37]. MiRNAs are encoded in the genome and originate from partly double-stranded RNA precursors. Mature miRNAs are released by the consecutive action of two RNase III enzymes, DROSHA and DICER [25]. DROSHA processes long primary transcripts (pri-miRNAs) into defined pre-miRNA hairpins. Then Dicer releases the mature miRNA duplex, and assists loading one of the strands into an AGO-clade Argonaute protein. Like all RNase III enzymes, DROSHA and DICER generate dsRNA products with characteristic 5’ monophosphates and 3’ 2-nucleotide overhangs. The 5’ monophosphate is a hallmark of all small silencing RNAs and provides key interactions with its Argonaute protein partner [38,39]. It also presents unhindered access for 5’ to 3’ exoribonucleases, and results in rapid degradation of these small RNAs in the absence of their Argonaute protein partner.

PiRNAs originate from single-stranded RNA-precursors and hence their biogenesis does not depend on RNase III enzymes [40]. The single-strandedness of their precursors, and the resulting requirement for different processing factors are distinctive features of piRNAs, and discriminate them from siRNAs and miRNAs. PiRNA generating genomic regions -‘piRNA clusters’ – are defined by mapping piRNA sequences to the genome and identifying extended genomic intervals that produce multiple piRNAs [41–43]. Some of these clusters span hundreds of kilobases in length and produce millions of different piRNAs. Whether these genomic regions are transcribed into a contiguous long transcript or produce multiple overlapping precursor RNAs remains largely unknown. The large fraction of multimapping reads derived from transposon fragments either within or outside these clusters complicates the genomic analysis of piRNAs and their precursor transcripts in flies and mammalian pre-pachytene piRNAs. However, the mammalian-specific class of pachytene piRNAs -named after their peak occurrence during the pachytene stage of meiotic prophase I – comprises mostly unique-mapping sequences, most of which are thought to target little but their own genomic origin [44,45].

### PiRNA generating genomic regions and piRNA precursors

The unique sequence space of pachytene piRNAs facilitated thorough characterization of their precursor transcripts. Pachytene piRNA-precursors are often driven by bidirectional promoters and comprise about 240 long single stranded transcripts with constitutive splicing patterns [46]. Knock-out experiments revealed phenotypes for two major piRNA precursors on chromosome 18 (pi18) and 6 (pi6) respectively [47,48]. Like knock-out of PIWL1/MIWI, the major PIWI protein partner during meiosis, knock-out of either piRNA cluster resulted in male sterility. However, while PIWIL1/MIWI null germ cells arrest at the round spermatid stage and never enter spermiogenesis, loss of piRNA-precursors pi18 and pi6 exhibit defects during later stages of sperm maturation. Surprisingly, elimination of the most prolific piRNA-generating region on chromosome 17 (pi17) remained without phenotype [47,49]. These data suggest that pachytene piRNAs might function redundantly, and knock-out of multiple piRNA-generating regions is required to reveal a complete phenotype. Another intriguing hypothesis suggests that some piRNA precursors might have developed a life of their own and become ‘selfish’ like their ancestral targets [45].

Similar to pachytene piRNA-precursors in mice, the function of individual *Drosophila* piRNA-clusters remains largely unknown. To date, only two essential piRNA clusters, Flamenco (Flam) and Suppressor of Stellate (Su(Ste)), have been discovered [50–52]. Flamenco has long been known as a major transposon control region in ovaries and is required for transposon restriction and female fertility [52]. The Y-linked Su(Ste) locus produces piRNAs that silence the X-linked Stellate genes to ensure male fertility [53]. Surprisingly, knock-out of another three major piRNA clusters −42AB, 20A and 38C- remained without phenotype [54]. Redundancy and overabundance of piRNAs, or piRNA production by dispersed transposable elements in cis have been proposed to ensure transposon restriction in the absence of individual piRNA clusters in the *Drosophila* ovary.

Little is known about what determines a piRNA-generating region, and what marks precursor transcripts for processing into piRNAs. Non-canonical transcription initiation, and splicing suppression enable transcription of dual-stranded piRNA clusters in flies [55], and mouse pachytene piRNA-genes require the transcription factor A-Myb [46]. RNA sequence and structure motifs, transacting factors, ribosome-occupancy, and piRNA-guided slicing were shown to licence piRNA-production from long precursor transcripts, but a universal mechanism remains elusive [56–62]. One could envision that individual piRNA precursors use different or a combination of signals and adaptors. Alternatively, it might be the absence of another identification that destines a transcript for fragmentation into piRNAs. Further studies are required to better understand piRNA precursors and the mechanisms that licence piRNA production.

### 5’ end processing and formation of pre-piRNAs

**Figure 3.**
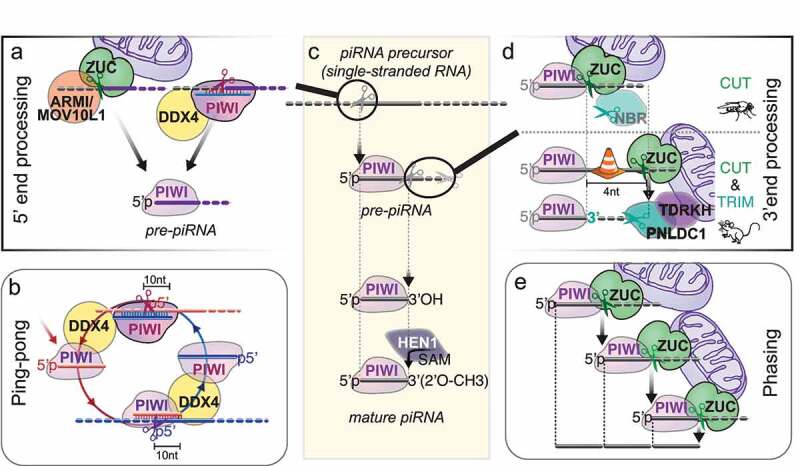
PiRNA biogenesis in flies and mice. (A) 5’ monophosphorylated (5'p) RNA fragments are generated either by the endonuclease Zucchini/PLD6 (ZUC) or by PIWI’s piRNA-guided slicing activity. ZUC collaborates with the RNA helicase Armitage (ARMI)/MOV10L1 to generate primary piRNAs on the surface of mitochondria. PiRISC collaborates with the germ cell-specific helicase Vasa/MVH/DDX4 (DDX4). (B) During ping-pong, coordinated slicing amplifies piRNA pairs with ten nucleotide complementarities. piRNA-guided slicing (‘ping’) generates a 5’ monophosphorylated RNA fragment that is loaded into another PIWI protein with the help of the RNA helicase Vasa/MVH/DDX4 (DDX4) to form a new piRISC (‘pong’). (C) piRNA biogenesis overview. Single stranded piRNA precursors are cleaved to generate 5’ monophosphorylated RNA fragments that are loaded into a PIWI protein to generate pre-piRNAs. 3'end processing proceeds on the PIWI-pre-piRNA complex and is completed by 2’-O-methylation by the methyltransferase HEN1/Pimet (HEN1) to produce mature piRNAs. (D). 3’ end processing. In *Drosophila*, most mature 3’ ends are generated by a single endonucleolytic cleavage by the ZUC-processor complex. The 3’-5’ exonuclease Nibbler (NBR) trims some ping-pong piRNAs but is not required for piRNA function. In mice, ZUC cleaves the pre-piRNA four nucleotides away from what is suggested to be a PIWI footprint, and 3’ end processing proceeds with obligatory 3’-5’ trimming by the exonuclease PNLDC1/Trimmer. 3’ end processing occurs on the surface of mitochondria. ZUC is directly hooked into the outer mitochondrial membrane. PNLDC1 is recruited to the surface of mitochondria by the Tudor protein TDRKH. (E) Phased piRNAs are produced by consecutive ZUC-cleavages.

Fragmentation of long RNA precursors and loading of RNA fragments into PIWI proteins to generate PIWI-interacting RNAs (piRNAs) happen on or near the surface of mitochondria and require either the endonuclease ZUCCHINI/PLD6/MITOPLD(ZUC) or piRNA-guided slicing (Figure 3(a, b)) [4,10]. ZUC belongs to a conserved family of HKD-phosphodiesterases that comprises RNases, DNases and phospholipases [63]. The substrate-specificity of these enzymes is determined by their substrate-binding surface, which folds into positively charged grooves for nucleases and potted structures to accommodate phospholipid head-groups for phospholipases [64]. In contrast to Dicer’s specificity for double-stranded RNA, ZUC specifically recognizes single-stranded substrates [64,65]. But like Dicer and piRNA-guided slicing, ZUC generates products with 5’ monophosphates, a prerequisite for stable association with Argonaute proteins. ZUC was originally identified in a screen for female sterility in *Drosophila*, and later implicated in piRNA biogenesis in flies and mice [66–69]. Structure-function studies revealed that ZUC itself was the nuclease required for primary piRNA biogenesis [64,65,70].

ZUC generates primary piRNAs that preferentially start with a Uridine (U) in the 5’ most position [4]. This **1 U-bias** has long been observed for piRNAs associated with Piwi and Aub in flies, and PIWIL1/MIWI and PIWIL2/MILI in mice [42,71,72]. The preference for Uridine in the 5’ most position is established during 5’ end formation by the ZUC-processor complex and reinforced upon Piwi binding [73]. Additional sequence preferences were attributed to the ZUC-processor complex in silkworm but have so far not been observed in flies or mice [74]. It remains unknown whether sequence preference during piRNA processing is a function of the ZUC nuclease itself or is established by a co-factor. A key component of the ZUC-processor complex is the conserved 5’ to 3’ RNA helicase Armitage (Armi)/MOV10L1 [69,75,76]. Armi/MOV10L1 is sufficient to induce primary piRNA biogenesis when tethered to a reporter construct [59], and knock-out of MOV10L1 or mutations of the catalytic helicase domain resulted in sterile animals [77]. Armitage/MOV10L1 marks transcripts for piRNA production, has the potential to remodel the ZUC-processor, and contributes to the formation of pre-piRNA complexes. ZUC itself is anchored to the surface of the mitochondria, and primary 5’ end formation, piRISC loading and 3'end formation proceeds on the outer mitochondrial membrane. The contribution of lipid interactions and membrane dynamics hamper simple biochemical purifications of the ZUC-processor and piRISC-loading complexes, and our understanding of piRNA biogenesis remains rudimentary. Novel methodologies based on proximity-ligation and chemical-crosslinkers promise routes towards understanding piRNA biogenesis and piRISC formation [78].

**3'end formation of piRNAs** occurs on assembled PIWI-pre-piRNA complexes and determines the piRNA length (Figure 2(c, d)). The early observation that different PIWI proteins associate with piRNAs with distinct length profiles suggested that piRNA length might reflect a footprint of the associated PIWI protein [42]. In *Drosophila*, the ZUC-processor complex generates most mature 3'ends [62,79]. In silkworm cells and in mice, 3’ termini undergo additional exonucleolytic trimming [80–83]. Both trimmed and untrimmed piRNAs exhibit a single major 3'end that is determined by hierarchical length and sequence preferences [79]. Conserved patterns in fly and mouse piRNAs suggested that the associated PIWI protein restricts accessibility for the ZUC-processor. Within the accessible 3’ zone, the Uridines positions the major ZUC-processing site. 3’ end formation faces an additional roadblock in mice that positions the initial ZUC cleavage site four nucleotides downstream of the mature 3'end and establishes opportunity for exonucleolytic trimming. PNLDC1 functions as piRNA-Trimmer in mouse and silkworm and is required for male fertility [80–83]. The Tudor protein TDRKH/ PAPI tethers PNLDC1 to the outer mitochondrial membrane and is required for piRNA 3’ maturation [80,82,84]. An unrelated exonuclease, Nibbler (Nbr), trims a fraction of piRNAs in flies but is dispensable for fertility [85,86]. Finally, most mature piRNAs are methylated at the 3'end by the methyltransferase HEN1/Pimet [87–89]. Only the mysterious group of mammalian PIWIL3-associated piRNAs remains unmethylated [12,90].

### Secondary piRNA biogenesis during ‘ping-pong’

Coordinated target-slicing and PIWI- loading generates secondary piRNAs during ‘ping-pong’ [42,91] (Figure 2b). PIWI-piRNA complexes bind target RNAs with extensive complementarity, and base-pairing across nucleotide 10 and 11 of the guide RNA enables PIWI’s nuclease activity to slice the target. Slicing generates products with 5’ monophosphates that can be loaded into PIWI proteins with the help of the germ cell specific helicase DDX4/VASA/MVH [92,93]. The helicase activity of DDX4 is required for this feed-forward amplification of piRNA-pairs [92]. In embryonic male germ cells in mice, PIWIL2/Mili ‘ping-pongs’ with itself and loads secondary piRNAs into PIWIL4/Miwi2, that then enter the nucleus to establish lasting epigenetic restriction of transposons [94]. The slicer activity of PIWIL2/Mili but not PIWIL4/Miwi2 is required for fertility [95]. In adult germ cells, the Tudor protein RNF17 suppresses secondary piRNA production [96]. In the absence of RNF17, untimely ping-pong could generate piRNA-pairs with potentially unwanted off-target effects, and the sterility of RNF17 mice was suggested to reflect an RNA-based autoimmune pathology.

In *Drosophila* germ cells, Aub and Ago3 engage in ping-pong that is initiated by Aub-piRISC (ping) and generates secondary Ago3-piRNAs [42,91,97]. Directionality of this heterotypic ping-pong is coordinated by the Tudor protein Qin/Kumo [98,99]. Ago3-piRNAs preferentially contain an Adenine (A) in position ten (‘10A’) that has been suggested to result from direct interaction with amino acids residues of its generating Aub-piRISC [100]. This Adenine in position ten could in turn generate the 1 U-bias of consecutive ping-pong piRNAs in Aub. In contrast, Uridine in the first position (1 U) cannot be responsible for the 10A-bias because the first nucleotide of small silencing RNAs is universally buried in their Argonaute protein partner and not available for base-pairing [101]. 1 U- and 10A-preferences are associated with specific piRNA-populations, and we are starting to understand the molecular mechanisms that establish these preferences. However, their impact on piRNA function remains largely elusive.

### Phased piRNAs – ‘inchworming’

Additional processing patterns include ‘phasing’, also called ‘inchworming’. When the last nucleotide of a piRNA directly neighbours the start of the next piRNA, and the 3'end of this piRNA is next to the 5'end of another one, we observe a phased pattern of piRNAs [57,61,62]. The coincidence of 5’ and 3’ ends of neighbouring piRNAs could either reflect preferential cleavage sites on different precursor transcripts, or simultaneous generation of a 3’ and a 5’ end by a single ZUC-cleavage event. The latter hypothesis is supported by an additional signature that was first observed in rhino (rhi) mutant ovaries in *Drosophila* [62]. Rhino (HP1d) is paralog of the Heterochromatin Protein 1 (HP1) and required for transcription of bidirectional piRNA clusters [55,102,103]. In the absence of rhino, secondary piRNAs aberrantly cleave mRNAs and induce a trail of phased piRNAs from the target mRNA (JB Sci15). Such ping-pong-induced trailing piRNAs were to some extent also observed in wild-type flies, and suggest additional communication between post-transcriptional and transcriptional silencing mechanisms in flies. Recent data from the Aravin lab suggest that siRNA silencing complexes also induce piRNA-production and contribute to the lasting establishment of piRNA-generating regions [104]. Ping-pong and phased piRNA production can be conceptually compared to secondary siRNAs in nematodes and plants [34,105]. These organisms contain RNA-dependent RNA polymerases (RdRPs), that amplify and extend siRNA repertoires. In brief, a primary siRNA-silencing complex recruits an RdRP to a complementary target RNA and produces double stranded RNAs that is diced into secondary siRNAs. Drosophila and mammals do not express RNA-dependent RNA polymerases, and ‘ping-pong’ and ‘phasing’ might have developed as alternatives to amplify and extend a primary piRNA signal. Further improvements of bona-fide piRNA-sequencing methods and bioinformatic tools are bound to reveal additional patterns, and improve our understanding of this diverse class of small RNAs [106–112].

### Cellular abundance is key to function in piRNA guided silencing

Sequence diversity is a hallmark of piRNAs. How these millions of diverse piRNAs faithfully silence their targets and avoid off-target effects remains an outstanding question in genome biology. If every piRNA we ever detected was to silence a target in every cell, piRNAs would silence a large fraction of the genome and degrade most transcripts. However, not every piRNA sequence functions in target restriction. We recently showed that the cellular abundance of piRNAs is key to piRNA-guided silencing [112]. The abundance of individual piRNA sequences is highly skewed and ranges over three to four orders of magnitude in flies and mice. Only the topmost abundant piRNAs are reproducible in different biological experiments. These robust piRNAs comprise the majority of all piRNA molecules but only a small fraction of the diverse sequences. A model emerges that categorizes piRNAs by sequence abundance. The topmost abundant piRNAs control silencing. PiRNAs of intermediate abundance are not present in every cell but could generate reproductive polymorphism, especially when they converge on a target RNA. The abundance of individual piRNAs is regulated by the identity of their precursor and by processing preferences, and we are just starting to uncover rules that govern the functional piRNA sequence space. Most low-abundant piRNAs are only detected sporadically and never contribute to target restriction. However, the extensive sequence space of these sporadic piRNAs provides opportunity for evolutionary tinkering and might enable adaptation to control novel genomic invaders.

### The challenge to recognize novel genomic invaders and establish adaptive immunity

Most piRNA studies investigate the adaptive restriction of resident transposons. Studies on unleashed transposons in dysgenic fly crosses revealed that maternally contributed PIWI-piRNA complexes induced lasting restriction of the escapist/runaway and regained genome stability [113]. However, little is known about the initial germ cell response to a novel genomic invader. A seminal study in wild Koalas observed an unexpected first response to a novel retrovirus by the piRNA pathway [114]. This innate response -without prior knowledge of the virus sequence- specifically identified the viral RNA genome and fragmented it into ‘sense’ piRNAs. This is analogous to antiviral defence mechanisms in plants, whereby DICER degrades viral double stranded RNA into siRNAs [115]. However, in contrast to siRNA processing that simultaneously degrades the virus and generates ‘antisense’ guides, the produced sense-piRNAs cannot mount an adaptive response. While the fragmentation of the viral genome by the piRNA processing machinery might be able to halt the viral infection temporarily, it requires an antisense transcript from an integrated provirus to initiate an adaptive response. Moving forward, it will be important to understand how piRNA pathways recognize novel viruses and initiate an innate response and how adaptive RNA-based immunity is successfully established. The exciting data from the current retroviral endogenization in Koala showed that gaining control over a novel genomic parasite is possible, but it also illustrates painfully that this process comes at a high price. Throughout evolution, our genomes have suffered conflict, accumulated scars, and established control. Many failed attempts have been eliminated from the genetic pool. What we observe today is the success story of RNA-based immunity.
